# A comparison of clinical characteristics of psychiatric inpatients in three hospitals from Western China and America

**DOI:** 10.1186/s12888-022-04500-2

**Published:** 2023-01-03

**Authors:** Min Jia, Bang An, Bin Yan, Qingyan Ma, Binglong Wen, Shanshan Zhao, Chengge Gao, Xiancang Ma, Lili Zhang, Bin Li, Ping Zhang, Jian Wang, Hong Yu, Wei Wang

**Affiliations:** 1 Department of Psychiatry, The First Affiliated Hospital of Xi’an Jiaotong University, 277 Yanta West Road, Xi’an, 710061 China; 2 Department of Psychiatry, Xianyang Central Hospital, 78 Renmin East Road, Xianyang, 712099 China; 3 Clinical Research Center for Psychiatric Medicine of Shaanxi Province, The First Affiliated Hospital of Xi’an Jiaotong University, 277 Yanta West Road, Xi’an, 710061 China; 4 Department of Sleep Medicine, Hebei Mental Health Center, 572 Dongfeng East Road, Baoding, 050899 China; 5 University at Buffalo-Psychiatry, Erie County Medical Center, 462 Grider Street, Buffalo, New York USA

**Keywords:** Psychiatry ward, Clinical characteristics, China, America

## Abstract

**Background:**

Different countries have differences in social and cultural context and health system, which may affect the clinical characteristics of psychiatric inpatients. This study was the first to compare cross-cultural differences in the clinical characteristics of psychiatric inpatients in three hospitals from Western China and America.

**Methods:**

Overall, 905 and 1318 patients from three hospitals, one in America and two in Western China, respectively, were included. We used a standardised protocol and data collection procedure to record inpatients’ sociodemographic and clinical characteristics.

**Results:**

Significant differences were found between hospitals from the two countries. Positive symptoms were the main reason for admission in the Chinese hospitals, while reported suicide and self-injury symptoms more frequently led to hospital admission in America. Moreover, there were more inpatients with combined substance abuse in the American hospital (97.6% vs. 1.9%, *P <* 0.001). The length of stay (LOS) in America was generally shorter than in China (10.5 ± 11.9 vs. 20.7 ± 13.4, *P <* 0.001). The dosage of antipsychotic drugs used in the American hospital was higher than in China (275.1 ± 306.9 mg vs. 238.3 ± 212.5 mg, *P* = 0.002). Regression analysis showed that male sex, older age, retirees, being admitted because of physical symptoms, and using higher doses of antipsychotic drugs were significantly associated with longer hospitalisation in the American hospital (*P <* 0.05). Comparatively, patients who were divorced, experiencing suicidal ideation, admitted involuntarily, admitted because of physical, depression, or anxiety symptoms, and using higher doses of antipsychotic drugs had longer hospitalisation in Chinese hospitals (*P <* 0.05).

**Conclusion:**

Significant variations in clinical characteristics of inpatients were found between hospitals from Western China and America. The LOS in Chinese hospitals was significantly longer, but patients used higher doses of antipsychotic drugs in the American hospital. Admission due to physical symptoms and the use of higher dosage drugs were related to longer LOS in both countries.

**Supplementary Information:**

The online version contains supplementary material available at 10.1186/s12888-022-04500-2.

## Introduction

In recent decades, mental health service systems in China and America have made substantial advances with economic and cultural development [1, 2]. Different cultural backgrounds, management systems, treatment patterns, and other factors can affect clinical characteristics of psychiatric inpatients.

The Chinese Mental Health Survey in 2019 showed that the prevalence of most mental disorders has increased; the weighted lifetime prevalence of six major types of mental disorders, excluding dementia, was 16.6%. The most common disorders were anxiety disorders (7.6%), followed by mood disorders (7.4%), substance use disorders (4.7%), impulse control disorders (1.5%), and schizophrenia and other psychotic disorders (0.7%) [3]. The above results also indicate that the prevalence of schizophrenia remains globally unchanged [4]. Comparatively, the survey of American adolescents showed that anxiety disorders were the most common condition (31.9%), followed by behaviour (19.1%), mood (14.3%), and substance use (11.4%) disorders; the comorbidity rate that meets the diagnosis of both mental disorders was approximately 40% [5]. The above survey emphasized that we should shift our attention from the treatment of American youth to prevention and early intervention.

As a representative of western countries, America began to reduce the number of large psychiatric hospitals and transform them into community services many years ago. Furthermore, mental hospitals have been replaced by psychiatric outpatients at general hospitals [6]. With the development of this ‘deinstitutionalisation’, more patients return to society and recover better; however, there is also evidence that this deinstitutionalisation increases the risk of violent crime [7]. The mental health service system in China has also learned from the experiences of other developed countries. Consequently, after continuous efforts, the number of registered psychiatric doctors and psychiatric hospital beds has increased significantly. However, compared with the large population of patients in China, there is still a limited number of hospital beds [8]. Mental health services rely on mental health hospitals and are separate from the community. This model is not beneficial for the long-term development of patients and increases the burden on the country [9]. Since 2004, China has implemented a national community service model called the National Continuing Management and Intervention Program for Psychoses (686 Program) [1], that has helped patients receive systematic treatment and follow-up.

However, both countries differ in their social and cultural context, health system, and clinical practice, which may affect the clinical characteristics of psychiatric inpatients. To date, no studies have compared the medical information of psychiatric inpatients between hospitals from Western China and America. The aim of this study was thus to address this gap by comparing the differences in clinical characteristics of psychiatric inpatients from three hospitals in these two countries, to provide some references for further research.

## Methods

### Research design and subjects

The Chinese information was obtained from the psychiatric department of two tertiary hospitals in Shaanxi Province: The First Affiliated Hospital of Xi’an Jiao Tong University and Xianyang Central Hospital, both of which are general hospitals. The American data were obtained from a general hospital with a psychiatric ward located at Buffalo, University at Buffalo-Psychiatry, Erie County Medical Center. All three hospitals have established hospital information systems, from which major information on clinical characteristics was obtained.

Data were collected by reviewing medical records. Using a form designed specifically for this study, information was collected between January and December 2015 from inpatients who were hospitalised in these hospitals. Sociodemographic information included gender, age, marital status, education level, occupation, and living conditions. Medical information mainly comprised reasons for hospitalisation, which was determined by several experienced experts on the basis of review and discussion of previous medical records (If a patient was admitted with plural reasons, the most prominent symptom was defined as the reason for hospitalization), whether they were admitted voluntarily, whether they were restrained or isolated during hospitalisation, existing condition with substance abuse, suicidal ideation, insurance, diagnosis with International Classification of Diseases Tenth Revision (ICD-10) or The Diagnostic and Statistical Manual of Mental Disorders Fifth Edition (DSM-5), the diagnosis was confirmed by at least two well-trained psychiatrists, and the length of stay (LOS) and antipsychotic drug dosage (the dosage here refers to the final stable daily dosage). Antipsychotic dosages were converted into chlorpromazine equivalent milligrams (CPZeq) [10, 11]. Owing to the anonymous nature of the retrospective chart review and for the purpose of clinical review, informed consent was not required, which was consistent with local ethical standards; only the medical records were reviewed. The study was approved by the Medical Ethics Committees of these hospitals.

### Statistical analyses

All statistical analyses were performed using SPSS 24.0. A t-test was used to compare continuous variables, a chi-square test and Fisher’s exact test were used to compare categorical variables. First, univariable factor linear regression analysis was used to screen variables affecting LOS. Then, the variables with statistical significance in the univariable regression analysis were included into new multivariable linear regression models to analyze variables affecting LOS in Western China and America, respectively. Moreover, the one-sample Kolmogorov-Smirnov test was used to check the normality of the distribution for the continuous variables. All statistical tests were two-sided, and a *P*-value < 0.05 was considered statistically significant.

## Results

### Socio-demographic characteristics of psychiatric inpatients

Inpatients’ socio-demographic characteristics in China and America are summarised in Table 1. A total of 2223 patients’ data, 905 from the American hospital and 1318 from the Chinese hospitals, were reviewed. In America, the proportion of male patients in psychiatric hospitals (62.1%) was significantly higher than females (37.9%). Comparatively, in China, the proportion of female patients (57.1%) was higher than male patients (42.9%). The proportion of unmarried inpatients in America was the highest (76.7%), while nearly half of the inpatients in the Chinese hospitals were married (59.1%), which accounted for the highest proportion. Patients with high school education levels were the highest in both countries (56.8% in America and 34.4% in China). The proportion of unemployed patients in the American (84.4%) was higher than in Chinese hospitals (11.2%). In both countries, most patients lived with their families; however, the proportion in the Chinese hospitals was higher than in America (99.7% vs 70.7%, *P <* 0.001).

**Table 1:** Socio-Demographic of psychiatric inpatients in western China and America

	America (***N*** = 905)	China (***N*** = 1318)	***t/χ2***	***P***
**Age (years,** $\overline{x}$ **±*s*)**	39.7 ± 13.7	40.3 ± 18.6	0.906	0.365
**Gender,** ***n*** **(%)**			78.772	<0.001
Male	562 (62.1%)	566 (42.9%)		
Female	343 (37.9%)	752 (57.1%)		
**Marital status,** ***n*** **(%)**			498.645	<0.001
Unmarried	694 (76.7%)	463 (35.1%)		
Married	112 (12.4%)	779 (59.1%)		
Divorce	79 (8.7%)	44 (3.3%)		
Widowed	20 (2.2%)	32 (2.4%)		
**Education,** ***n*** **(%)**			429.311	<0.001
Primary school and below	7 (0.8%)	238 (18.1%)		
Junior middle school	36 (4.0%)	355 (26.9%)		
High school	514 (56.8%)	453 (34.4%)		
Junior college or undergraduate	348 (38.5%)	272 (20.6%)		
**Occupation,** ***n*** **(%)**			1276.030	<0.001
Workers	106 (11.7%)	227 (17.2%)		
Freelance worker	5 (0.6%)	496 (37.6%)		
Students	10 (1.1%)	181 (13.7%)		
Retiree	20 (2.2%)	267 (20.3%)		
The unemployed	764 (84.4%)	147 (11.2%)		
**Living condition,** ***n*** **(%)**			423.615	<0.001
Living alone	265 (29.3%)	4 (0.3%)		
Groups life	640 (70.7%)	1314 (99.7%)		

*N* total number of subjects in the study cohort; *n* (%) number (percentage) of patients with the indicated characteristic

### Clinical characteristics of psychiatric inpatients

Table 2 presents the clinical characteristics of the psychiatric inpatients. The reasons for admission differed, with suicide and self-injury symptoms accounting for the largest proportion in the American hospital (46.1%), which was much higher than in China (7%), followed by positive symptoms (44.9%). In the Chinese hospitals, the main reason for admission was positive symptoms (46.3%). Most patients were admitted involuntarily in America (94.1%), and voluntarily (64.5%) in China. Moreover, the proportions of patients who were restrained or isolated in these two countries were below 15% (13.7% in America and 13.1% in China, *P* = 0.657). Compared with China, there were more inpatients with combined substance abuse in America (97.6% vs 1.9%, *P <* 0.001). Among them, 54.3% of patients combined alcohol and drug abuse and 43.3% combined narcotics. In the Chinese hospitals, only 1.9% of patients had combined substance abuse and no inpatients had combined narcotics. In both countries, more than 90% of patients had insurance (98.6% in America and 99.3% in China, *P* = 0.078).

**Table 2:** Clinical characteristics of psychiatric inpatients in western China and America

	America (***N*** = 905)	China (***N*** = 1318)	***t/χ2***	***P***
**Reasons for admission,** ***n*** **(%)**			622.279	<0.001
Positive symptoms	406 (44.9%)	610 (46.3%)		
Negative symptoms	6 (0.7%)	67 (5.1%)		
Depression and anxiety	43 (4.8%)	379 (28.8%)		
Suicide and self-injury	417 (46.1%)	92 (7.0%)		
Physical symptoms	18 (2.0%)	157 (11.9%)		
Substance abuse	9 (1.0%)	8 (0.6%)		
Drug side effects	6 (0.7%)	5 (0.4%)		
**Whether or not to be admitted voluntarily,** ***n*** **(%)**			764.821	<0.001
Yes	53 (5.9%)	850 (64.5%)		
No	852 (94.1%)	468 (35.5%)		
**Whether restrained or isolated,** ***n*** **(%)**			0.197	0.657
Yes	124 (13.7%)	172 (13.1%)		
No	781 (86.3%)	1146 (86.9%)		
**Substance abuse,** ***n*** **(%)**			2034.822	<0.001
None	22 (2.4%)	1293 (98.1%)		
Substance abuse	883 (97.6%)	25 (1.9%)		
Alcohol and Drugs	491 (54.3%)	25 (1.9%)		
Narcotics	392 (43.3%)	0 (0.0%)		
**Suicidal ideation,** ***n*** **(%)**			2.807	0.094
Yes	466 (51.5%)	631 (47.9%)		
No	439 (48.5%)	687 (52.1%)		
**Insurance,** ***n*** **(%)**			3.110	0.078
Yes	892 (98.6%)	1309 (99.3%)		
No	13 (1.4%)	9 (0.7%)		
**Diagnosis,** ***n*** **(%)**			183.445	<0.001
Organic mental disorder	29 (3.2%)	252 (19.1%)		
Mental and behavioral disorders due to psychoactive substance use	41 (4.5%)	10 (0.8%)		
Schizophrenia Spectrum Disorder	341 (37.7%)	454 (34.4%)		
Mood disorder	326 (36.0%)	345 (26.2%)		
Depression	176 (19.4%)	199 (15.1%)		
Bipolar disorder	150 (16.6%)	146 (11.1%)		
Neurotic, stress-related and somatoform disorders	160 (17.7%)	221 (16.8%)		
Behavioral syndrome with physiological disorders and somatic factors	1 (0.1%)	16 (1.2%)		
Personality and behavioral disorders	6 (0.7%)	2 (0.2%)		
Intellectual disability	1 (0.1%)	7 (0.5%)		
Mental development disorder	0 (0.0%)	11 (0.8%)		
**LOS (days,** $\overline{x}$ **±*s*)**	10.5 ± 11.9	20.7 ± 13.4	18.719	<0.001
Organic mental disorder	9.9 ± 18.1	20.2 ± 12.3	4.023	<0.001
Mental and behavioral disorders due to psychoactive substance use	7.3 ± 5.4	13.9 ± 5.3	3.519	0.001
Schizophrenia Spectrum Disorder	13.8 ± 13.8	22.4 ± 16.0	7.983	<0.001
Mood disorders				
Depression	8.6 ± 8.9	19.7 ± 10.1	11.251	<0.001
Bipolar disorder	10.3 ± 8.1	23.3 ± 16.0	8.842	<0.001
Neurotic, stress-related and somatoform disorders	6.8 ± 11.9	17.1 ± 8.0	10.129	<0.001
Behavioral syndrome with physiological disorders and somatic factors	9.0 ± 0.0	17.3 ± 8.7	0.926	0.369
Personality and behavioral disorders	8.7 ± 5.1	13.5 ± 10.6	0.932	0.387
Intellectual disability	30.0 ± 0.0	23.3 ± 5.3	−1.189	0.279
Mental development disorder	–	21.3 ± 16.9	–	–
**Total dose of antipsychotics (mg,** $\overline{x}$ **±*s*)**	275.1 ± 306.9	238.3 ± 212.5	−3.129	0.002
Organic mental disorder	133.0 ± 152.1	155.6 ± 135.1	0.840	0.402
Mental and behavioral disorders due to psychoactive substance use	153.2 ± 191.7	261.0 ± 228.5	1.537	0.131
Schizophrenia Spectrum Disorder	447.2 ± 329.0	386.2 ± 216.3	−2.973	0.003
Mood disorder				
Depression	77.0 ± 124.5	117.7 ± 108.7	3.383	<0.001
Bipolar disorder	364.8 ± 296.0	280.2 ± 212.9	−2.831	0.005
Neurotic, stress-related and somatoform disorders	99.0 ± 189.2	118.8 ± 156.1	1.118	0.264
Behavioral syndrome with physiological disorders and somatic factors	420.0 ± 0.0	120.1 ± 109.2	−2.665	0.018
Personality and behavioral disorders	222.5 ± 240.1	300.0 ± 424.3	0.340	0.746
Intellectual disability	510.0 ± 0.0	285.7 ± 163.2	−1.285	0.246
Mental development disorder	–	169.6 ± 139.4	–	–

*N* total number of subjects in the study cohort; *n* (%) number (percentage) of patients with the indicated characteristic

Moreover, schizophrenia was the most evident diagnosis in both countries (37.7% in America and 34.4% in China), followed by mood disorders (36.0% in America and 26.2% in China). The proportion of organic mental disorder patients in Chinese hospitals was higher than in America (19.1% vs 3.2%, *P <* 0.001) but that of psychoactive substances in America was higher than in China (4.5% vs 0.8%, *P <* 0.001). Patients diagnosed with behavioural syndrome with physiological disorders and somatic factors, personality and behavioural disorders, intellectual disability, and mental development disorders were less than 5% in both countries. Owing to the few patients diagnosed with these diseases, they were excluded when comparing the LOS. Overall, the LOS in hospitals in America was shorter than in China (10.5 ± 11.9 vs 20.7 ± 13.4, *P <* 0.001). Individuals with schizophrenia had the longest hospitalisation in America (13.8 ± 13.8), followed by bipolar disorder (10.3 ± 8.1). In China, the longest hospitalisation was for bipolar disorder (23.3 ± 16.0), followed by schizophrenia (22.4 ± 16.0). In general, excluding the above-mentioned four diseases, the total dosage of antipsychotic drugs used in the American hospital was higher than in China (275.1 ± 306.9 mg vs 238.3 ± 212.5 mg, *P* = 0.002). In America, the dosage of antipsychotic drugs in patients with schizophrenia spectrum disorder (447.2 ± 329.0 mg vs 386.2 ± 216.3 mg, *P* = 0.003) and bipolar disorder (364.8 ± 296.0 mg vs 280.2 ± 212.9 mg, *P* = 0.005) was higher than in China. However, it is worth noting that the dosage of antipsychotic drugs used by patients with unipolar depression in Chinese hospitals was higher than in America (117.7 ± 108.7 mg vs 77.0 ± 124.5 mg, *P <* 0.001). Overall, patients with schizophrenia had the largest dosage of antipsychotic drugs in both countries (447.2 ± 329.0 mg in America and 386.2 ± 216.3 mg in China), followed by bipolar disorder (364.8 ± 296.0 mg in America and 280.2 ± 212.9 mg in China).

### Factors affecting LOS

Table 3 presents the regression analysis results in the American hospital. In univariable analysis, we found that nine variables were significantly associated with the LOS. We then conducted a multivariable regression analysis of these variables and found that males, older patients, retirees, patients admitted because of physical symptoms, and patients who used higher dosages of antipsychotic drugs had significantly longer LOS (*P <* 0.05). Stratified by gender, we found that male inpatients had more severe symptoms and took higher doses drugs (Supplementary Table).

**Table 3:** Mixed linear regression model testing associations of predictors with LOS in American hospital

Variables	Univariable linear regression				Multivariable linear regression			
	β	Cl(95%)		***P***	β	Cl(95%)		***p***
		Lower bound	Upper bound			Lower bound	Upper bound	
**Age**	0.143	0.087	0.199	<0.001	0.144	0.082	0.205	<0.001
^**a**^**Gender**	−2.945	−4.536	−1.354	<0.001	−2.078	−3.635	−0.521	0.009
^**b**^**Marital status**								
Unmarried	2.503	0.124	4.881	0.039	1.656	−0.670	3.982	0.163
Divorce	2.321	−1.111	5.753	0.185	1.201	−1.993	4.396	0.461
Widowed	1.575	−4.095	7.245	0.586	−1.018	−6.322	4.287	0.707
^**c**^**Education**								
Junior middle school	4.056	−5.578	13.689	0.409				
High school	3.325	−5.550	12.199	0.462				
Junior college or undergraduate	1.253	−7.650	10.156	0.782				
^**d**^**Occupation**								
Workers	−14.471	−20.097	−8.844	<0.001	−10.596	−16.171	−5.021	<0.001
Freelance worker	−17.250	− 28.789	− 5.711	<0.003	− 11.526	− 22.429	−0.622	0.038
Students	−15.750	− 24.688	−6.812	0.001	− 10.967	−19.761	− 2.173	0.015
The unemployed	−12.028	−17.256	−6.801	<0.001	−9.761	−14.956	−4.566	<0.001
^**e**^**Living condition**								
Groups life	0.901	−0.807	2.608	0.301				
^**f**^**Substance abuse**								
Yes	−5.139	−10.176	−0.102	0.046				
^**g**^**Suicidal ideation**								
Yes	4.001	2.467	5.534	<0.001	−0.434	−3.493	2.626	0.781
^**h**^**Insurance**								
Yes	6.471	−0.050	12.991	0.052				
^**i**^**Whether or not to be admitted voluntarily**								
Yes	−1.206	−4.516	2.104	0.475				
^**j**^**Whether restrained or isolated**								
Yes	3.524	1.275	5.773	<0.002	0.379	−1.958	2.716	0.751
^**k**^**Reasons for hospitalization**								
Negative symptoms	2.584	−6.805	11.972	0.589	5.493	−3.439	14.425	0.228
Depression and anxiety	−1.649	−5.310	2.012	0.377	1.466	−2.194	5.125	0.432
Suicide and self-injury	−4.138	−5.730	−2.546	<0.001	−0.609	−3.858	2.639	0.713
Physical symptoms	10.195	4.696	15.694	<0.001	9.720	4.515	14.926	<0.001
Substance abuse	−7.750	−15.443	−0.056	0.048	−4.810	− 12.410	2.790	0.215
Drug side effects	−6.416	−15.805	2.972	0.180	−4.936	−13.946	4.074	0.283
^**l**^**Diagnosis**								
Organic mental disorder	−3.878	−8.287	0.531	0.085	0.108	−4.311	4.528	0.962
Psychoactive substance	−6.541	−10.309	−2.773	0.001	−2.659	−6.352	1.034	0.158
Mood disorder								
Depression	−5.241	−7.357	−3.126	<0.001	−1.070	−3.636	1.496	0.413
Bipolar disorder	−3.523	−5.756	−1.289	0.002	−1.696	−3.883	0.492	0.129
Neurotic, stress-related and somatoform disorders	−6.972	−9.156	−4.788	<0.001	−2.627	−5.205	−0.048	0.046
Behavioral syndrome with physiological disorders and somatic factors	−4.809	−27.637	18.018	0.679	−1.416	−23.230	20.398	0.899
Personality and behavioral disorders	−5.143	−14.530	4.245	0.283	−0.069	−9.122	8.983	0.988
Intellectual disability	16.191	−6.637	39.018	0.164	12.694	−8.841	34.228	0.248
Mental development disorder	–							
**Total dose of antipsychotics**	0.012	0.009	0.014	<0.001	0.010	0.007	0.013	<0.001

^a^Reference category = Male; ^b^Reference category = Married; ^c^Reference category = Primary school and below; ^d^Reference category = Retiree; ^e^Reference category = Living alone; ^f^Reference category = None substance abuse; ^g^Reference category = No suicidal ideation; ^h^Reference category = No insurance; ^i^Reference category = Admitted involuntarily; ^j^Reference category = No restrained or isolated; ^k^Reference category = Positive symptoms; ^l^Reference category = Schizophrenia Spectrum Disorder

Table 4 shows the factors affecting LOS in Chinese hospitals. We conducted multivariable regression with seven variables that were statistically significant in the univariable analysis and found that patients who were divorced, had suicidal ideation, were admitted involuntarily, were admitted because of physical, depression or anxiety symptoms, and who used greater dosages of antipsychotic drugs had longer hospitalisations (*P <* 0.05). Collinearity analysis showed that there was no obvious collinearity among all factors included in the multivariable linear model for America and China.

**Table 4:** Mixed linear regression model testing associations of predictors with LOS in Chinese hospital

Variables	Univariable linear regression				Multivariable linear regression			
	β	Cl(95%)		***P***	β	Cl(95%)		***p***
		Lower bound	Upper bound			Lower bound	Upper bound	
**Age**	−0.006	− 0.045	0.033	0.750				
^**a**^**Gender**	−1.066	−2.527	0.395	0.152				
^**b**^**Marital status**								
Unmarried	1.533	0.002	3.063	0.050	1.248	−0.428	2.924	0.144
Divorce	9.052	5.010	13.094	<0.001	7.209	3.352	11.067	<0.001
Widowed	−0.616	−5.320	4.089	0.797	−2.154	−6.611	2.303	0.343
^**c**^**Education**								
Junior middle school	1.634	−0.567	3.835	0.145				
High school	1.537	−0.566	3.640	0.152				
Junior college or undergraduate	0.745	−1.586	3.077	0.531				
^**d**^**Occupation**								
Workers	1.589	−0.783	3.961	0.189				
Freelance worker	−0.125	−2.119	1.870	0.902				
Students	0.367	−2.163	2.897	0.776				
The unemployed	0.580	−2.119	3.278	0.674				
^**e**^**Living condition**								
Groups life	2.167	−10.990	15.324	0.747				
^**f**^**Substance abuse**								
Yes	−5.443	−10.741	−0.146	0.044	−4.063	−10.239	2.113	0.197
^**g**^**Suicidal ideation**								
Yes	5.921	4.508	7.334	<0.001	3.801	1.926	5.676	<0.001
^**h**^**Insurance**								
Yes	5.363	0.231	−3.420	0.231				
^**i**^**Whether or not to be admitted voluntarily**								
Yes	−7.193	−8.655	−5.732	<0.001	−4.759	−6.765	−2.753	<0.001
^**j**^**Whether restrained or isolated**								
Yes	1.454	−0.693	3.601	0.184				
^**k**^**Reasons for hospitalization**								
Negative symptoms	−4.583	−7.943	−1.222	0.008	−1.744	−4.948	1.460	0.286
Depression and anxiety	−1.102	−2.810	0.606	0.206	2.653	0.396	4.911	0.021
Suicide and self-injury	−4.126	−7.046	−1.206	0.006	1.441	−1.901	4.783	0.398
Physical symptoms	−3.221	−5.558	−0.885	0.007	4.323	1.382	7.265	0.004
Substance abuse	−10.941	−20.232	−1.650	0.021	−1.916	− 13.965	10.133	0.755
Drug side effects	1.859	−9.865	13.583	0.756	0.549	−10.469	11.568	0.922
^**l**^**Diagnosis**								
Organic mental disorder	−2.258	−4.301	− 0.216	0.030	−2.619	−5.150	−0.087	0.043
Psychoactive substance	−8.545	−16.858	−0.232	0.044	−0.289	−11.717	11.140	0.960
Mood disorder								
Depression	−2.756	−4.967	−0.546	0.015	−2.737	−5.668	0.193	0.067
Bipolar disorder	0.891	−1.583	3.365	0.480	−0.790	−3.320	1.741	0.541
Neurotic, stress-related and somatoform disorders	−5.305	−7.438	− 3.172	<0.001	−3.032	− 5.819	− 0.244	0.033
Behavioral syndrome with physiological disorders and somatic factors	−5.132	−11.747	1.482	0.128	−3.973	−10.817	2.871	0.255
Personality and behavioral disorders	−8.945	−27.373	9.483	0.341	−4.602	− 22.010	12.806	0.604
Intellectual disability	0.841	−9.063	10.745	0.868	2.011	−7.350	11.372	0.674
Mental development disorder	−1.172	−9.107	6.763	0.772	−1.244	−8.837	6.349	0.748
**Total dose of antipsychotics**	0.013	0.010	0.017	<0.001	0.014	0.010	0.018	<0.001

^a^Reference category = Male; ^b^Reference category = Married; ^c^Reference category = Primary school and below; ^d^Reference category = Retiree; ^e^Reference category = Living alone; ^f^Reference category = None substance abuse; ^g^Reference category = No suicidal ideation; ^h^Reference category = No insurance; ^i^Reference category = Admitted involuntarily; ^j^Reference category = No restrained or isolated; ^k^Reference category = Positive symptoms; ^l^Reference category = Schizophrenia Spectrum Disorder

## Discussion

To our knowledge, this is the first study comparing the clinical characteristics of inpatients with psychiatric disorders in hospitals from Western China and America. Our results found differences in the sociodemographic and clinical characteristics of psychiatric inpatients. Most hospitalised patients were men in the American hospital and women in the Chinese hospitals. Furthermore, there were more unmarried and unemployed inpatients in the American hospital. The highest proportion of reasons for admission was suicide and self-injury symptoms in America and positive symptoms in China. Compared with China (1.9%), there were more inpatients with combined substance abuse in the American hospital (97.6%). The two most common diseases in hospitalised patients in these two countries were schizophrenia and mood disorders. Moreover, the LOS in the American hospital was shorter, but the total dosage of antipsychotic drugs was significantly higher than in the Chinese hospitals.

With regards to the higher percentage of psychiatric inpatients for substance use in America, it was noted that the proportion of patients diagnosed with psychoactive substances in American hospitals was also higher than that in China. There is a consensus that the incidence of mental disorders and comorbid substance abuse is very high [12–14]. To illustrate, it is estimated that, in America, the lifetime prevalence of patients with mental disorders combined with some addictive disorder was 29.0%, alcohol dependence was 13.5%, and other drug dependence abuse was 6.1%. Moreover, 37% of those with an alcohol disorder had comorbid mental disorders. In addition, more than half (53%) of patients with drug addiction (excluding alcohol) had a mental disorder. Comorbidity rates of severe mental disorders, such as schizophrenia, bipolar disorder, and anti-social personality disorder, are particularly high, which makes treatment challenging [15]. In China, the most common use of psychoactive substances is drinking alcohol, with most other psychoactive substances being illegal and banned. Moreover, men are more likely to use alcohol than women [16, 17]. To illustrate, alcohol is widely used in China because a drinking culture of repetitive toasting plays a huge role in building relationships with business partners and friends; however, the rate may decrease due to the recent anti-corruption campaign in China which banned the provision of alcoholic beverages in government-sponsored meals [18]. However, the use of some psychoactive substances, such as cannabis, is legal in some states of America. Many states in America have legalised either its medical or its recreational use, making it easier to obtain. This leads to a marked increase in the prevalence of cannabis use, especially among subgroups [19–22]. The above factors may explain why more psychiatric inpatients combined substance abuse. Furthermore, this highlights the need to focus on inpatients who are diagnosed with psychoactive substances in America in the future. Suicidal behaviour is a leading cause of injury and death worldwide. We found that the proportion of patients admitted because of attempted suicide and self-injury in America was higher than in China. The World Health Organization report indicates that in developed countries, suicide accounts for the largest share of intentional injury burden and the proportion of men is higher than women [23]. Risk factors for suicide include demographic, psychiatric, psychological, and stressful life events. Men with mental disorders, especially mood disorders and substance abuse, are at high risk for suicide [24]. Consistent with this, our results showed that these factors are higher in the American hospital.

Our data also showed that psychiatric LOS in America was significantly shorter than in China, but the total dosage of antipsychotic drugs was significantly higher. The LOS for mental disorders has decreased in America in recent decades [25]. These reductions have coincided with the reduction of public mental health services and an expansion in care provided in private psychiatric and general hospitals [26, 27]. Inpatient care constitutes the most expensive part of psychiatric services. To reduce expenditure, inpatients are required to be discharged as soon as possible [28]. The Healthcare Cost and Utilisation Project on mental health stays in American community hospitals in 2006 showed that the average LOS for mental health hospitalisation was less than 10 days [29]. Consistent with this, a recent report determined that the average LOS in an American inpatient setting was 10.0 ± 3.0 days [30]. Our results confirmed that the average LOS in an American hospital was 10.53 ± 11.91 days. There is consensus that psychotropic drugs usually take effect after 2–4 weeks [31–34]. In China, after being discharged from the hospital, patients rely on family members to take care of them. These family members lack professional knowledge, making it difficult for them to observe improvement in symptoms and adverse drug reactions; thus, patients require a longer hospital stay. Although a reduction in LOS can reduce expenditure, it may lead to early discharge of patients who remain clinically unstable [35]. Furthermore, some studies have claimed that the shorter the hospital stay, the higher the relapse and readmission rate [36]. Therefore, more research is needed to determine which service system is more beneficial to patients. However, with updates to the guidelines, the requirements for rehabilitation of social function with mental illnesses have increased, and most patients may need to recover in the community in the future. Therefore, in China, the improvement of community services will also be a major challenge.

Furthermore, our results showed that the total dosage of antipsychotic drugs used in the American hospital was higher than in China. Ethnic differences in the dosage of antipsychotic drugs have been confirmed by many studies; Caucasians usually require higher antipsychotic dosages than Asians [37–40]. The differential response and side effect profile of antipsychotics are related to genetic makeup, individual intrinsic factors, and cultural and contextual variables [41–44]. Some data show that Caucasians are more likely to have received treatment for all psychiatric disorders (including bipolar disorder, anxiety, and substance use disorders) and have regular follow-ups [45]. Some articles have demonstrated lower clearance in the average East Asian population using antipsychotics due to metabolic activities [40]. These results may be because the dosage of drugs used in America is higher than in China. We found that there was a combination of antipsychotics inpatients with depression in both countries. In recent years, some studies have demonstrated that combining antidepressants and antipsychotics is significantly more effective to treat psychotic depression than using either antidepressant or antipsychotic monotherapy [46]. Most treatment guidelines recommend a combination of an antidepressant with an antipsychotic to treat an acute episode of unipolar psychotic depression [47]. Therefore, antipsychotic drugs are usually used in patients with depression, especially those with psychotic depression. Our results show that inpatients with depression in China used higher dosage of antipsychotics. It is indicated that there is more drug combination in China.

We also analysed the factors affecting the LOS in these two countries. The results showed that admission due to physical symptoms and the use of high-dosage drugs were related to longer LOS. In America, males also had longer hospitalisations, the LOS for male patients is longer, which may be related to the presence of more severe symptoms and higher drugs doses in male inpatients. In China, except for the above factors, patients who were divorced, had involuntary admissions, and experienced suicidal ideation had longer lengths of stay. Almost all studies have achieved consensus that patients with schizophrenia have the longest hospital stay among psychiatric conditions; this is because patients who suffer from severe mental illnesses need lengthier stays to achieve stabilisation and partial recovery [48, 49]. According to previous studies, older patients often have longer hospital stays due to the nature of their physical diseases [50–52]. Comparatively, married patients have shorter stays because they usually have better emotional support and social networks, and family members, who can help patients to regularly take their medication and adhere to treatment plans [53]. In addition, due to the psychosocial nature of mental disorders, relatives’ active participation can enable patients to recover better. This may explain why unmarried and divorced patients had longer hospital stays in this study. Moreover, organic comorbidities are often accompanied by psychiatric problems and patients usually need consultations to reach a better clinical state, which leads to an increase in hospital stay [54, 55]. Patients in psychiatric wards who are restrained and isolated are usually those who have aggressive, impulsive, or violent behaviours; are less compliant with treatment procedures; and have negative attitudes towards medication. Additionally, physicians are more cautious in assessing the discharge of patients who may harm others; thus, longer hospitalisation is required to overcome these barriers [56]. This is consistent with the results of our study.

The main strength of this study is that it is the first to compare the clinical characteristics of psychiatric inpatients in these two countries in the East and West. In addition, the use of comprehensive clinical information, yielded significant results. Moreover, all information was collected through routine practice, which was not biased by any specific research protocol. These strengths ensure that the results reasonably reflect real-world situations. However, this study also had several limitations that should be highlighted. First, this was a retrospective study, and the data were collected by chart review, which might have led to observational bias. Second, we only selected three general hospitals from these two countries, thus, this study’s results should be interpreted with caution. Third, the type and name of other drugs were not obtained, such as mood stabilizers or anti-depressants, and only the dosages of chlorpromazine equivalent milligrams of antipsychotic drugs were included.

In conclusion, this study’s results highlighted the differences in the clinical characteristics of inpatients with psychiatric disorders in hospitals from Western China and America. Therefore, these findings serve as a starting point for determining the reasons behind these differences in future studies.

## Supplementary Information

**Additional file 1: Supplementary Table.** Clinical characteristics between male and female inpatients in America hospital.

## Data Availability

The datasets used and/or analyzed during the current study are available from the corresponding author (Wei Wang) on reasonable request.

## Abbreviations

ICD-10: International Classification of Diseases Tenth Revision
DSM-5: The Diagnostic and Statistical Manual of Mental Disorders
CPZeq: Chlorpromazine equivalent milligrams
LOS: Length of stay
