# Supplementary material for: A comparison of clinical characteristics of psychiatric inpatients in three hospitals from Western China and America
Source: BMC Psychiatry. 2023 Jan 3;23:6. doi: 10.1186/s12888-022-04500-2 (PMC9811694; doi:10.1186/s12888-022-04500-2)
Supplement: Supplementary file 1 — Additional file 1: Supplementary Table. Clinical characteristics between male and female inpatients in America hospital. [file 12888_2022_4500_MOESM1_ESM.docx]

**Supplementary Table** Clinical characteristics between male and female inpatients in America hospital

|  | **Male**  **(N=562)** | **Female**  **(N=343)** | ***t/χ2*** | ***P*** |
| --- | --- | --- | --- | --- |
| **Age** | 38.8±13.7 | 41.3±13.7 | -2.68 | 0.01 |
| **LOS (days, $\bar{x}$±*s*)** | 11.7±12.6 | 8.7±10.4 | 3.81 | ＜0.001 |
| **Total dose of antipsychotics (mg, $\bar{x}$±*s*)** | 313.9±323.7 | 211.6±265.6 | 5.17 | ＜0.001 |
| **Occupation, n (%)** |  |  | 1.460 | 0.853 |
| Workers | 71 (12.6%) | 35 (10.2%) |  |  |
| Freelance worker | 3 (0.5%) | 2 (0.6%) |  |  |
| Students | 6 (1.1%) | 4 (1.2%) |  |  |
| Retiree | 12 (2.1%) | 8 (2.3%) |  |  |
| The unemployed | 470 (83.6%) | 294 (85.7%) |  |  |
| **Reasons for admission, n (%)** |  |  | 34.292 | ＜0.001 |
| Positive symptoms | 292 (52.0%) | 114 (33.2%) |  |  |
| Negative symptoms | 3 (0.5%) | 3 (0.9%) |  |  |
| Depression and anxiety | 18 (3.2%) | 25 (7.3%) |  |  |
| Suicide and self-injury | 230 (40.9%) | 187 (54.5%) |  |  |
| Physical symptoms | 9 (1.6%) | 9 (2.6%) |  |  |
| Substance abuse | 6 (1.1%) | 3 (0.9%) |  |  |
| Drug side effects | 4 (0.7%) | 2 (0.6%) |  |  |
